# Integrating women’s voices in quality improvement for maternity care: A qualitative study

**DOI:** 10.18332/ejm/152253

**Published:** 2022-09-05

**Authors:** Evelien Cellissen, Maaike Vogels-Broeke, Irene Korstjens, Marianne Nieuwenhuijze

**Affiliations:** 1 Research Centre for Midwifery Science, University of Midwifery Education and Studies, Zuyd University of Applied Science, Maastricht, Netherlands; 2 Care and Public Health Research Institute (CAPHRI), Maastricht University, Maastricht, Netherlands

**Keywords:** qualitative research, maternity care, patient experience, woman-centered care, healthcare quality improvement

## Abstract

**INTRODUCTION:**

Improving the quality of maternity care is high on the national agenda in the Netherlands. One aspect gaining significant attention is integrating women’s experiences – as users of maternity care – in this quality improvement. The aim of this study was to gain deeper insights into how maternity care professionals in Dutch Maternity Care Collaborations integrate women’s voices into quality improvement as part of integrated maternity care and what role midwives can have in this.

**METHODS:**

This was a descriptive qualitative study, using semi-structured individual interviews and content analysis for an in-depth exploration of maternity care professionals’ experiences and opinions on integrating women’s voices in quality improvement. Participants were twelve maternity care professionals involved in quality improvement activities from eight Dutch Maternity Care Collaborations.

**RESULTS:**

Four themes emerged: ‘Quality improvement based on women's voices is still in its infancy’ and was experienced as an important but challenging topic; ‘Collecting women's voices’ was conducted, but needed more facilitation; Using women's voices’ was hindered by a lack of expertise and a structured feedback and feedforward system; and ‘Ensuring listening to women's voices’ and integrating them in quality improvement required further facilitation.

**CONCLUSIONS:**

Care professionals emphasized that listening to women’s voices for quality improvement is important but challenging due to the lack of expertise, organizational structure, time, and financial resources. A feasible implementation strategy including concrete support is recommended by maternity care professionals to boost action.

## INTRODUCTION

Twenty years ago, the US Committee on the Quality of Health Care recommended six aims for improving the healthcare system. One important aim was that healthcare in the 21st century should be patient-centred^1^. A strategy for stimulating patient-centeredness is the collection of data on patient’s experiences and satisfaction for the purpose of quality improvement^2,3^.

To achieve quality improvement in healthcare, it is essential to move towards measuring, reporting, and comparing patient experiences^4-6^. This move is also visible in maternity care. The content of women’s experiences is conditional to improve quality of care. Downe et al.^7^ emphasized the need for moving from what professionals tend to think from their perspective is important to women, towards what women actual find is most important to them in maternity care^7^. Through collecting their experiences, the quality of care can be continuously improved.

The World Health Organization (WHO) recommendations for antenatal, intrapartum and postpartum care emphasize women’s experiences of maternity care as meaningful and necessary to establish woman-centered care^8-10^. In 2016, to enhance a woman-centered approach in Dutch maternity care, the various professional organizations in maternity care jointly developed a national standard, the Integrated Maternity Care Standard^11^. This Care Standard addresses the need for a safe, effective, and woman-centered maternity care system with closer collaboration between maternity care professionals through integrated care organized around women. The Care Standard sets the norm for how to organize prenatal, natal and postnatal care and how the collaboration between maternity care professionals should be organized including the implementation of a quality system. Maternity Care Collaborations (MCCs) are accountable for implementing the care standard and improving regional quality of care^12^. Over the past decade, these MCCs have been established in many regions across the country and include at least regional maternity care services, such as a hospital, independent midwifery practices and organizations of maternity care assistants^13^.

One of the challenging aspects of the Care Standard is the implementation of a cross-organizational quality system for the region, for which each MCC is responsible^11^. This cross-organizational system must include women’s experiences of their care and should give all professionals (e.g. midwives, obstetricians) within an MCC insight into how pregnant women experience integrated care during pregnancy, birth and the postnatal period, and how it can be improved. In this quality system, the woman as user of maternity care is central rather than the organization or the professional.

The nationwide dissemination of the Care Standard did not automatically result in the implementation of this quality system in the MCCs^14^. The aim of this study was to gain deeper insights into how maternity care professionals in MCCs integrate women’s voices into quality improvement as part of the Integrated Maternity Care Standard and what role midwives can have in this.

## METHODS

A qualitative, descriptive study with individual interviews allowed an in-depth exploration of motives, experiences, and opinions of care professionals on integrating women’s voices into quality improvement within MCCs. As integrating women’s voices in quality improvement is rather new in the Netherlands, this might be a sensitive topic for participants feeling uncertain about the uptake of their new task. Also, existing (hierarchical) positions between midwives, managers and obstetricians might influence the participants’ responses. Therefore, individual interviews were more likely to offer them the safety to reveal their true motives^15^.

### Setting and participants

For the present study, care professionals were asked how they used women’s voices to improve quality of care within their MCC using reports provided as part of the StEM-study. The StEM-study is a research project to explore the preferences and experiences of women who give birth in the Netherlands. Two care professionals per MCC (22 professionals in total) were invited, each having some experience with quality improvement in their MCC. We sought for variety in work experience, backgrounds (profession, gender, age), professional roles (midwife, manager, obstetrician), and levels of expertise implementing quality management. In the invitation e-mail, we informed them that participation was voluntary, that their information would be handled confidentially, and that data would be securely stored at the university digital network with only the research team having access.

The StEM study was approved by the Medical Research Ethics Committee METC Z, Heerlen (METCZ20180121). Women, recruited through eleven MCCs across the Netherlands, filled in surveys that included several validated instruments on women’s experiences, such as Nijmegen Continuity Questionnaire (NCQ)^16^, Birth Satisfaction Scale (BSS)^17^, and Mother’s Autonomy in Decision Making Scale (MADM)^18^. The Integrated Maternity Care Standard^11^ obliges MCCs to measure women’s experiences using validated instruments and use this for quality improvement. By participating in the StEM-study MCCs could meet this obligation. Each of the participating MCCs received a report with the anonymized results of women’s experiences in their MCC. In our study, purposive sampling was used among the MCCs participating in the StEM-study^19^.

### Data collection

Between June and September 2020, individual interviews were conducted with the participants lasting 45–60 minutes using videoconferencing because of COVID-19 restrictions. We used a semi-structured question route (Table 1) based on literature about quality improvement in healthcare and patient involvement^2,3,20^.

**Table 1:** Examples from the semi-structured question route

How do you think women’s experience can be implemented in quality improvement?
What items do you find important to hear from women?
How did you use the data from the report?
How do you use quality data from women to improve quality of care?

The first author, experienced in maternity care, policy advising, and quality management, conducted all interviews. The last author, experienced in qualitative research in maternity care, participated in the first two interviews and provided feedback. After each of the first three interviews, the question route was refined. All participants received their transcripts, five responded that they agreed with the transcript without further remarks, the other participants did not react.

### Analysis

A content analysis was performed using manual inductive and deductive coding to identify themes and patterns between the themes^15,19,21^. Subthemes were grouped into main themes by examining the commonalities, differences, and relationships within and among the interviews, and through reflective discussion among the research team consisting of the authors and student assistant researchers. After reading and rereading all transcripts and coding one interview, the first author developed an initial coding tree that was advanced together with the last author based on the data of three, randomly chosen, interviews. Next, the first author and student assistant researchers independently coded the other transcripts. The research team refined the coding tree several times and reached consensus about the final coding tree. Saturation was reached after ten interviews, which was confirmed by the last two interviews. Our findings are illustrated by quotes, which were translated using backward and forward translation. Participants are indicated with a letter, without naming their profession for anonymity reasons.

### Rigor and reflectivity

To ensure trustworthiness, we followed the strategies recommended by Korstjens and Moser^22^. The research team was not professionally involved with the participants, and combined experience and expertise in qualitative research, maternity care, and quality management. We kept a logbook, including field notes from the interviews and the reflective discussions in the research team of organizational, scientific, and analytic processes. We identified quotes, which were translated backward and forward, assisted by a native English speaker. To secure anonymity, we present the quotes without reference to the participant’s professional background. The standards for reporting qualitative research (COREQ) guided the writing^15^.

## RESULTS

Twelve participants from eight MCCs agreed to participate. Midwives represent the largest group of six people in total. Furthermore, two obstetricians, and four managers in close contact with the workplace were included in this study. This mirrors the distribution of professionals within the workgroups of MCCs, such as a workgroup for quality improvement. Two participants identified as male and ten as female, varying in age (33–58 years) and work experience (3–28 years). The four themes that emerged from the analysis are listed in Table 2.

**Table 2:** Themes that emerged from the analysis

Quality improvement based on women’s voices in its infancy
Collecting women’s voices
Feedback and feedforward: using women’s voices
Ensuring listening to women’s voices

### Quality improvement based on women’s voices in its infancy

All participants emphasized that integrating women’s experiences of care in quality improvement was a significant, but challenging topic. Most MCCs had a designated workgroup for quality improvement with care professionals, which devoted part of their time (beside their care duties) to developing and revising protocols, organizing perinatal audits and skills training. Some participants said they did not feel knowledgeable enough to structurally imbed women’s experiences in quality improvement:

*‘We have a quality workgroup that deals with protocols and audits and so on, but not with patient input so to speak … on policy level … what patients think about protocols and care pathways and how they experience care things like that … I don't know what that would look like in practice.’* (Participant F)

A few MCCs had imbedded women’s experiences in their quality system. In these MCCs, the quality workgroup used the collected data on experiences: the workgroup discussed the results, selected notable items, formulated actions for improvement, and presented a summary of their findings to the other care professionals in their MCC. These MCCs had a more formal organizational structure and more management experience. However, participants were unable to indicate whether going through the steps of the quality cycle led to actual improvements in the quality of care they offered:

*‘Well, we received the report with the results, we filtered out the most remarkable things: the real points for improvement and the things that were already very good. We translated the results into a kind of short analysis with points for improvement. These points were also included in our quality improvement plan and immediately converted into actions.’* (Participant I)

Although all participants acknowledged its importance, in most MCCs quality improvement based on women’s voices was still in its infancy.

### Collecting women’s voices

For most participants, a quantitative survey was a good start for collecting women’s voices and gaining insight into women’s experiences. However, some participants expressed that they wanted more in-depth qualitative information from women to understand what really matters to them. Most participants found it difficult to articulate what topics needed further qualitative exploration. The lack of experience with structural quality improvement and how to include women’s voices was indicated as a barrier. The will to collect women’s voices was present, but care professionals lacked knowledge on how to do this effectively:

*‘… I'm not particularly trained for this, I mean, I know I can ask people how they experienced the care, but … to make a good survey …’* (Participant F)

Several participants expressed a lack of self-confidence in interpreting quantitative data. For example, they did not know what to expect in terms of satisfaction and hesitated about whether an item should be marked as ‘this could be better’ or ‘good enough’. Younger midwives seemed more skilled but were less involved in quality improvement tasks. Therefore, the results of quantitative experience reports were often not used for quality improvement purposes:

*‘I don't know how to interpret this … is this good or is it bad … should we improve this item? Maybe you can explain it to me?’* (Participant C)

A mother council was mentioned as a valuable addition to surveys for receiving in-depth qualitative information. Setting up a mother council is part of the Integrated Maternity Care Standard, but not easy to implement according to the participants as organizational structure and time were lacking. Next to that, participants lacked knowledge and experience on how to implement a mother council:

*‘… in our MCC, we also discussed how to increase patient participation. It would be good to set up a mother council, but that's not easy to do.’* (Participant J)

### Feedback and feed-forward: using women’s voices

Reports with MCC specific data were shared with each MCC as a return for the participation in the StEM study. Our participants mentioned that structured follow-up in quality improvement activities was low after these reports became available. Only some MCCs planned a discussion with women or made an action plan together with all care professionals within their MCC:

*‘… some results were at the top of our mind for a while and then … not much action was taken on it … and after a while everybody had forgotten about it … and they went on with their normal business.’* (Participant H)

Some participants considered revealing results on women’s experience of care between professional groups within their MCC (for example between midwives and obstetricians) or between different MCCs as a sensitive issue, as this might reinforce a sense of competition. Others favored sharing each other’s results because this would provide insights into difference and could stimulate improving quality of care:

*‘Some practices think they are doing a good job, so they say: we can show our data to others. But some practices find it difficult to give insight into their data because they are afraid, they are being compared to others … and that benchmarking causes tension.’* (Participant G)

### Ensuring the use of women’s voices

Lack of time, financial resources, and expertise to interpret the results, were important barriers to collect and give meaning to women’s voices. Suggested solutions were involvement of external parties such as professional associations or parties funded by the government to support care professionals in the MCCs. Their task could consist of providing national, digital, validated surveys including mandatory questions for regional and national benchmarking, and optional questions to explore regional relevant topics. To actually use women’s voices to improve quality of care a ready-to-use report, written by the same external party that collected the data, was suggested as a helpful tool to support the care professionals. Preferably, the results in this report are presented as visual factsheets and infographics:

*‘… how to implement? Less effort and maximum result so let others give us the information we need and tell us what to do with it …’* (Participant L)

### Analytic findings

Overall, it seems that maternity care professionals are currently more focused on running and improving the quality of their own healthcare practice, rather than collaborating on a regional level in their MCC. For integrating women’s experiences in cross-organizational quality improvement, a shared, structured, and formally embedded MCC quality system is needed. Establishing such a cross-organizational system requires different competencies, which most care professionals do not yet possess. Midwives did not always feel competent as it was not part of their regular daily care duties nor was it an extensive part of their midwifery education program in the past. Some participants noted that more recently graduated midwives did develop some of these competencies during their education. However, these midwives often did not participate in implementation of innovations, such as a MCC quality system, as they were more focused on mastering their midwifery skills. These findings illustrate that a feasible implementation strategy, including a sound analysis of barriers and facilitators, should accompany the dissemination of national standards, such as the Integrated Maternity Care Standard, to make implementation successful. If not, care professionals opt for instrumental and limited approaches, such as seeking support from other parties and using random ready-made instruments.

## DISCUSSION

The aim of this study was to gain deeper insights into how maternity care professionals in MCCs integrate women’s voices into quality improvement as part of the Integrated Maternity Care Standard and what role midwives can have in this. As a way to improve the implementation of this aspect of the standard, participants suggested a survey, supplemented with qualitative approach, for collecting women’s experiences.

This preference is also visible in other healthcare domains. Acceptance by care professionals of the way in which client experiences are surveyed is necessary to actually use the data for quality improvement^3,20,23^. Merely collecting and reporting experiences is not sufficient to achieve improvement of care, integrating them into the quality improvement system is essential^20,24-26^. The care professionals in MCCs struggled with using women’s voices for quality improvement, because there was no formally embedded cross-organizational quality improvement system. The barriers they experienced such as lack of time, expertise and organizational structure also exist in other healthcare domains^20,25^.

To provide care professionals with more insights into what women would like them to know, care professionals and women should also be involved in the macro-level of an organization. This involvement of people in organizations is reflected in Arnstein’s ‘Ladder of citizen participation’^27^. This ladder shows how citizens can participate at different levels of organizations. Arnstein describes that some public institutions deny power to citizens and keep them on a lower level, she also shows how these levels can be increased. In our study, the involvement of women did not go beyond the level of consultation. To involve women in the macro-level of an organization asks from MCCs to enhance women’s involvement to partnership^20^.

Moving from consultation towards partnership calls for a culture change within the MCCs^20^, which requires a sense of urgency to become established^28^. For the care professionals, the national Integrated Maternity Care Standard^11^ created this sense of urgency as an external motivator. Next to this, a guiding team is conditional for the culture change^29^. This team should consist of care professionals and women themselves^20,25^. The guiding team could play a role in providing a convincing argument that change is needed and in showing that change is working: evidence of change gives motivation for change. Appealing to the intrinsic values of the care professionals (e.g. woman-centeredness, autonomy) and showing that the change has perceived worth for the care professional either personally or professionally are interventions to apply^30^. To facilitate MCCs in moving towards partnership, support from leaders and resources in terms of time, financial resources, and organizational structures are necessary^4,20^.

Next to the external motivator, care professionals also need expertise and motivation to implement women’s voices in quality improvement. The professionals in our study were aware that they lack expertise in this area, even though they had the will (intrinsic motivation) to move forward. They saw a possible solution in an instrumental approach by asking external parties to provide surveys or reports. However, studies demonstrate that setting up external feedback systems rarely achieves quality improvement^31,32^. Care professionals themselves must be motivated and skilled to engage in co-creation processes with their clients to reflect on what is important to clients in quality improvement. This requires a different mind-set on the part of the care professional. Stimulation of this motivation to take actions is missing in top-down implementation of standards. The lack of expertise can also be compensated by allowing more recently graduated midwives to play a significant role in cross-organizational activities. These midwives seemed to develop more competencies needed to establish a structured quality system in a MCC during their education. Because a team approach is one of the prerequisites for successful implementation of quality systems^20^, newly graduated midwives need the encouragement of the MCC in order to become more involved in quality improvement activities. This requires leadership and a culture that acknowledges the expertise of the midwife. In addition, this also calls for facilitating recently graduated midwives to use their skills in the field of quality management.

### Strengths and limitations

Integrating women’s voices in quality improvement in maternity care needs attention in many countries, in that sense the Netherlands is not unique. Although some countries or other medical fields are more advanced, others still seek ways to achieve this. A trend towards integration of various services in maternity care is increasingly seen in the Netherlands and in other countries^12,33^. This means integration of care of regular maternity services with other services such as psychological or social care, both involving various professionals working together but not being part of the same organization. In the Netherlands, a cross-organizational quality system is being introduced in maternity care. This cross-organizational system must include women’s experiences of their care and should give all professionals within an MCC insight into how pregnant women experience integrated care and how it can be improved. In this quality system, the pregnant woman is central rather than the organization or the professional. This article provides insight into what maternity care professionals need, to use women’s voices to improve integrated maternity care and to implement a cross-organizational quality system. Other strengths were that the participants, who were all involved in quality improvement, varied in professional and sociodemographic backgrounds. We reached saturation after ten interviews. No new analytical information arose after ten interviews suggesting that we attained maximum information on our topic^17^. We included a specific population as purposive sampling was used by approaching the 11 MCCs participating in the StEM study^17^. The included MCCs did not have a leadership role in integrating women’s experiences into quality improvement but were willing when facilitated by StEM. Their motivation for participating in StEM was largely based on the Integrated Maternity Care Standard^9^. According to Rogers’ theory of innovations^32^, these characteristics are specific for early and late majority groups in implementing innovations, representing 68% of the population and called the mainstream. As our sample is likely to represent the mainstream, our findings are relevant for a large group and other early adopters who might consider transferring these findings to their contexts. Another limitation of the study was that we did not interview women who are involved in quality improvement in some MCCs. This is a future area for exploration.

## CONCLUSIONS

Care professionals in Dutch MCCs emphasized that using women’s voices for quality improvement was important but challenging due to lack of expertise, organizational structure, time, and financial resources. An implementation strategy is needed to implement a quality system in a cross-organizational context. To facilitate implementation, the instrumental part, such as providing national, digital, validated surveys and a ready-to-use report, should be made available by external parties. Facilitating the instrumental part gives the care professional time to set up and implement the quality system within an MCC. This external support might also boost actions of care professionals for integrating women’s voices in quality improvement. Encouraging these actions is lacking in top-down implementation of standards and should be included more from the development of standards onward. Finally, an implementation process requires identifying which competencies are needed for particular tasks and who has those competencies. Appointing the right people, in this case recently graduated midwives, to crucial positions can facilitate successful implementation of women’s voices in maternity care quality management.

## Data Availability

The data supporting this research are available from the authors on reasonable request.
